# The leukocyte-stiffening property of plasma in early acute respiratory distress syndrome (ARDS) revealed by a microfluidic single-cell study: the role of cytokines and protection with antibodies

**DOI:** 10.1186/s13054-015-1157-5

**Published:** 2016-01-12

**Authors:** Pascal Preira, Jean-Marie Forel, Philippe Robert, Paulin Nègre, Martine Biarnes-Pelicot, Francois Xeridat, Pierre Bongrand, Laurent Papazian, Olivier Theodoly

**Affiliations:** 1 Adhésion et Inflammation, Université Aix-Marseille, INSERM U1067, CNRS UMR7333, 163 avenue de Luminy, Marseille, 13009 France; 2 Laboratoire d’Immunologie, Assistance Publique - Hôpitaux de Marseille, 147, boulevard Baille, F-13285 Cedx 05 Marseille, France; 3 Assistance Publique - Hôpitaux de Marseille, Hôpital Nord, Réanimation des Détresses Respiratoires et des Infections Sévères, 13015 Marseille, France; 4 Aix-Marseille Université, Faculté de médecine, URMITE UMR CNRS 7278, 13005 Marseille, France

## Abstract

**Background:**

Leukocyte-mediated pulmonary inflammation is a key pathophysiological mechanism involved in acute respiratory distress syndrome (ARDS). Massive sequestration of leukocytes in the pulmonary microvasculature is a major triggering event of the syndrome. We therefore investigated the potential role of leukocyte stiffness and adhesiveness in the sequestration of leukocytes in microvessels.

**Methods:**

This study was based on in vitro microfluidic assays using patient sera. Cell stiffness was assessed by measuring the entry time (ET) of a single cell into a microchannel with a 6 × 9–μm cross-section under a constant pressure drop (ΔP = 160 Pa). Primary neutrophils and monocytes, as well as the monocytic THP-1 cell line, were used. Cellular adhesiveness to human umbilical vein endothelial cells was examined using the laminar flow chamber method. We compared the properties of cells incubated with the sera of healthy volunteers (n = 5), patients presenting with acute cardiogenic pulmonary edema (ACPE; n = 6), and patients with ARDS (n = 22), of whom 13 were classified as having moderate to severe disease and the remaining 9 as having mild disease.

**Results:**

Rapid and strong stiffening of primary neutrophils and monocytes was induced within 30 minutes (mean ET >50 seconds) by sera from the ARDS group compared with both the healthy subjects and the ACPE groups (mean ET <1 second) (p < 0.05). Systematic measurements with the THP-1 cell line allowed for the establishment of a strong correlation between stiffening and the severity of respiratory status (mean ET 0.82 ± 0.08 seconds for healthy subjects, 1.6 ± 1.0 seconds for ACPE groups, 10.5 ± 6.1 seconds for mild ARDS, and 20.0 ± 8.1 seconds for moderate to severe ARDS; p < 0.05). Stiffening correlated with the cytokines interleukin IL-1β, IL-8, tumor necrosis factor TNF-α, and IL-10 but not with interferon-γ, transforming growth factor-β, IL-6, or IL-17. Strong stiffening was induced by IL-1β, IL-8, and TNF-α but not by IL-10, and incubations with sera and blocking antibodies against IL-1β, IL-8, or TNF-α significantly diminished the stiffening effect of serum. In contrast, the measurements of integrin expression (CD11b, CD11a, CD18, CD49d) and leukocyte–endothelium adhesion showed a weak and slow response after incubation with the sera of patients with ARDS (several hours), suggesting a lesser role of leukocyte adhesiveness compared with leukocyte stiffness in early ARDS.

**Conclusions:**

The leukocyte stiffening induced by cytokines in the sera of patients might play a role in the sequestration of leukocytes in the lung capillary beds during early ARDS. The inhibition of leukocyte stiffening with blocking antibodies might inspire future therapeutic strategies.

**Electronic supplementary material:**

The online version of this article (doi:10.1186/s13054-015-1157-5) contains supplementary material, which is available to authorized users.

## Background

Acute respiratory distress syndrome (ARDS) has been identified as a bilateral pulmonary inflammatory condition that follows direct or indirect lung injury [1]. Rigorous clinical management has not reduced the mortality of ARDS to <30 % [2], mainly owing to a lack of understanding of ARDS pathophysiology. The sequestration of leukocytes, particularly neutrophils, in the lung microvasculature [3] appears to be a key determinant of the pathophysiology of ARDS, leading to blood circulation blockage, microthrombus formation [4, 5], uncontrolled inflammation, and injury to the alveolar–capillary membrane [1]. Researchers have attempted to clarify the mechanisms involved in leukocyte sequestration in many studies. Changes in leukocyte adhesion to vessel walls have been reported in various inflammation contexts [6–9], notably in ARDS [9]. In vitro studies [10, 11] have also shown that a fraction of circulating leukocytes of patients with ARDS, as well as patients with sepsis, trauma, and pneumonia, resulted in impaired deformability most likely caused by the densification of F-actin in the cortical region [6, 10], suggesting a role of leukocyte stiffening in sequestration of leukocytes in lung capillaries [4]. However, the roles of leukocyte adhesion and stiffness remain unclear. Microcirculation impairments have been associated with abnormal concentrations of cytokines and endotoxins in various inflammatory diseases [8–19], but the case of ARDS has rarely been studied. Moreover, consensus conclusions have been difficult to draw due to the diversity of experimental conditions and models. For instance, some studies have failed to show any effect of interleukin (IL)-8 on leukocyte stiffness [12, 19], whereas others have found a pronounced effect [16]. Additionally, IL-8 and tumor necrosis factor (TNF)-α have been reported to promote adherence in some conditions [12], whereas no effects have been detected in other studies [8, 20]. Altogether, it appears that the triggering events of ARDS, mainly leukocyte sequestration in the lungs, remain largely obscure with regard to the mechanisms and biochemical signaling involved.

In the present study, using in vitro microfluidic methods, we investigated leukocyte stiffness and adhesiveness in response to incubation in the sera of patients with ARDS. The time required for a leukocyte to penetrate a synthetic microchannel allowed us to assess the stiffness of the leukocytes, whereas a laminar flow chamber assay was used to characterize their adhesiveness. The main objectives of this study were first to demonstrate that the sera of patients with ARDS induced leukocyte stiffening and then to identify the cytokine conditions that induce leukocyte stiffening and test protective treatments.

## Methods

### Patients and sera

This prospective, observational study was conducted in the medical intensive care unit (ICU) of a teaching hospital. The investigation was approved by the local ethics committee of the Assistance Publique-Hopitaux de Marseille. All consecutive mechanically ventilated patients presenting with ARDS, as defined according to the Berlin definition [21], were included when they were early in the course of the disease. Within 2 days of ARDS onset, the lower ratio of partial pressure of oxygen in arterial blood to fraction of inspired oxygen (PaO_2_/FiO_2_) was measured and used to classify the patients with ARDS according to the Berlin definition. Patients with PaO_2_/FiO_2_ ratios <200 mmHg were assigned to the moderate to severe ARDS group, and patients with a PaO_2_/FiO_2_ ratio between 200 and 300 mmHg were assigned to the mild ARDS group. Volume assist control ventilation was used with a tidal volume of 6–8 ml/kg of predicted body weight and a maximal plateau pressure of 32 cmH_2_O. The oxygenation goal was a pulse oximetry–measured arterial oxygen saturation of 88–95 % or a PaO_2_ of 55–80 mmHg, with the FiO_2_ and positive end-expiratory pressure adjusted as in the ARMA trial [22]. Five healthy volunteers from our medical department served as the healthy group. Six mechanically ventilated patients with acute cardiogenic pulmonary edema (ACPE) were also enrolled as the ACPE group. Septic shock (SS) was defined in accordance with the 2001 Society of Critical Care Medicine/European Society of Intensive Care Medicine/American College of Chest Physicians/American Thoracic Society/Surgical Infection Society International Sepsis Definitions Conference criteria [23]. Patients presenting with immunological treatment, neoplasia, and corticosteroid treatments were not included.

To assay IL-6, 1 ml of serum was obtained from systemic arterial blood samples taken during the usual medical care of patients in the ICU. Blood samples were obtained from patients with ARDS and patients with ACPE within the first 48 h following the onset of the lung insult, and the samples were stored at −80 °C. Serum samples were also obtained from five healthy volunteers and were stored at −80 °C.

### Investigation strategy

The changes in the distribution of cell stiffness induced in a leukocyte population by serum incubation were assessed by taking systematic measurements of the leukocyte entry times (ETs) into a microfluidic constriction. The stiffening effects of the sera of patients with ARDS were first tested on primary neutrophils and monocytes. Then THP-1 cells were used for a systematic study of sera from the healthy volunteers and the ACPE and ARDS groups. To identify the cytokines potentially involved in cell stiffening, we correlated the mean ET for all of the subjects in the three groups with the cytokine levels in their sera. Recombinant cytokines were further used to confirm and quantify the stiffening potential of the relevant cytokines. Finally, cytokine-blocking antibodies (Ab) were added to the sera to analyze the respective involvement of the cytokines in the stiffening effects of the patients’ sera and to test for protective effects against the pathological stiffening effects of the patients’ sera.

### Microfluidic protocol to characterize the distribution of cell stiffness

The protocol [24] is depicted in Figure S1 (see Additional file 1). Cells incubated in sera were directed into microfluidic circuitry by varying the height of the macroscopic reservoirs (Additional file 1: Figure S1a) connected to a microfluidic chip positioned on a microscope stage (Additional file 1: Figure S1b). The ET into a microfluidic constriction is known to be a good indicator of cell stiffness [14, 25]. However, devices with a single constriction have been found to be poorly adapted for cells of elevated stiffness because the mismatch between the rectangular cross-section of microfluidic channels and the spherical shape of the cells allows for flow leakage around the cells (Additional file 1: Figure S1c[i]) and induces poor ET reproducibility. The device used in this study had two constrictions in series, C1 and C2, of similar width (*W* = 6 μm) and with heights of *H*_*1*_ = 12 μm and *H*_*2*_ = 9 μm, respectively (Additional file 1: Figure S1d, e). A cell was first forced into constriction C1, where it occupied the entire cross-section of the channel and therefore prevented leaks in corners (Additional file 1: Figure S1c[ii]). Cell stiffness was then assessed by examining the cell’s passage from constriction C1 into the narrower constriction C2. The ET of each single cell was determined by examining video microscopy sequences as the time interval between the leading edge of the cell touching the entrance of constriction C2 and the trailing edge of the cell clearing the entrance of constriction C2 (Additional file 1: Figure S1c[iii], c[iv]).

### Microfabrication

Microfluidic devices were fabricated using standard soft lithography procedure [26]. A positive mold was created with SU-8–negative resins (MicroChem, Westborough, MA, USA) on silicon wafers (Sil’Tronix, Archamps, France). Replicas were made in polydimethylsiloxane elastomer (SYLGARD® 184; Dow Corning, Auburn, MI, USA) and were sealed on glass cover slides via plasma activation (Harrick Plasma, Ithaca, NY, USA). The channels were incubated with a 1 % Pluronic F 108 solution (BASF, Florham Park, NJ, USA) for 1 h to deter cell adhesion. Observations were made using an inverted microscope (Axio Observer 200; Carl Zeiss Microscopy, Oberkochen, Germany) equipped with a phase contrast objective (Plan-Neofluar 100×/1.30 Oil Ph3; Carl Zeiss Microscopy) and a Burle TC65 camera (Burle).

### Cell preparation

Primary monocytes and neutrophils were isolated from whole blood and were analyzed within 3 h. Whole blood was collected in heparin vials from healthy donors who provided informed consent. For cell stiffness measurements, polymorphonuclear cells and peripheral blood mononuclear cells (PBMCs) were separately isolated from whole blood by gradient density centrifugation using Histopaque-1077 solution (Sigma-Aldrich, St. Louis, MO, USA). Monocytes were separated from the PBMC fraction by negative selection using the Pan Monocyte Isolation Kit, human (Miltenyi Biotec, Bergisch Gladbach, Germany). Red blood cells were eliminated from the polymorphonuclear fraction via hypotonic lysis. The monocytic THP-1 cell line (American Type Culture Collection, Manassas, VA, USA) was maintained in Gibco RPMI 1640 medium (Life Technologies, Carlsbad, CA, USA) supplemented with 10 % fetal calf serum and 2 mM l-glutamine. Incubations of cells with patients’ sera were performed for 1 h at 10 %. Cytoskeleton inhibition was performed with latrunculin A (Molecular Probes, Eugene, OR, USA) at 3 μg/ml and nocodazole (Sigma-Aldrich) at 5 μg/ml for 30 minutes. For the cytometric analysis, we used CD3, CD14, and CD66b Ab to control the efficacy of T lymphocyte, monocyte, and neutrophil purification procedures. Neutrophils were isolated from whole blood by negative selection using the EasySep™ Direct Human Neutrophil Isolation Kit (STEMCELL Technologies, Vancouver, BC, Canada). Monocytes were isolated from PBMC fractions by negative selection using the Pan Monocyte Isolation Kit, human (Miltenyi Biotec). Lymphocytes were studied using the PBMC fraction.

### Monoclonal antibodies

The following adhesion function-blocking Ab against human adhesion molecules were used: anti-l-selectin (CD62L), anti-αL chain integrin (CD11a), anti-integrin β1 (CD29), anti-αM chain of Mac-1 integrin (CD11b), α4 chain of VLA-4 integrin (CD49d), and anti-β2 chain of lymphocyte function-associated antigen 1 integrin (CD18) (all from BioLegend, San Diego, CA, USA). To discriminate cells in the cytometry data, we also used Ab against CD3, CD14, and CD66b (BioLegend). The following Ab against cytokines were used: anti-IL8 and anti-IL10 (AbD Serotec, Raleigh, NC, USA) and anti-TNF-α (Janssen Biologics BV, Leiden, the Netherlands). We also used Ab for control experiments: anti-mouse immunoglobulin G1 (IgG1) (AbD Serotec), mouse IgG1 (eBioscience, San Diego, CA, USA), human polyclonal Ab (LFB Biomedicaments, Ulis, France), and goat IgG1 (AbD Serotec).

### Reagents

Enzyme-linked immunosorbent assay (ELISA) kits for testing human TNF-α, interferon (IFN)-γ, transforming growth factor-β1, IL-1β, IL-6, IL-8, IL-10, and IL-17 were obtained from R&D Systems (Minneapolis, MN, USA). Recombinants of IL-8, TNF-α, and IL-10 were purchased from Life Technologies, and recombinants of IL-1β were purchased from PeproTech (Rocky Hill, NJ, USA).

### Cytometric and adhesion measurements

Cytometric measurements were performed with a BD LSR II flow cytometer (BD Biosciences, San Jose, CA, USA). Labeling was performed according to the manufacturer’s recommendations. Adhesion between circulating leukocytes and model endothelium were examined in vitro using the laminar flow chamber technique and human umbilical vein endothelial cells (PromoCell, Heidelberg, Germany) [27]. Cells were pushed by horizontal flow and sedimented by gravity in the vicinity of the endothelial cell monolayer cultured to confluence on a glass substrate coated with fibronectin. The frequencies of cell arrests under constant hydrodynamic shear stress were monitored.

### Statistical analysis

The distribution was evaluated using the Kolmogorov-Smirnov test. According to the variable distributions, differences between groups were assessed using one-way analysis of variance (with post hoc Tukey’s test), Student’s *t* tests, the Mann-Whitney *U* test, Wilcoxon’s test, or Fisher’s exact test. The correlations between ETs and cytokine concentrations were assessed using Spearman’s test. A *p* value <0.05 was considered significant. All of the reported *p* values are two-sided. If not mentioned, statistical analysis was conducted using IBM SPSS version 20.0 software (IBM, Armonk, NY, USA). Graphs were created using GraphPad Prism software (GraphPad Software, La Jolla, CA, USA).

## Results

### Patients

Twenty-two patients who presented with early ARDS were included. Thirteen patients were classified in the moderate to severe ARDS group, and 9 patients were included in the mild ARDS group. Among patients with ARDS, 15 patients presented with associated SS. The patient characteristics are summarized in Table 1.

**Table 1:** Patient characteristics

Characteristics	Healthy (*n* = 5)	ACPE (*n* = 6)	Mild ARDS (*n* = 9)	Moderate/severe ARDS (*n* = 13)
Age, yr	38 [34–43]	72 [68–82]	57 [42–71]	58 [56–76]
Sex, M/F, *n*	2/3	3/3	4/5	9/4
Septic shock, *n*	–	–	5	10
SAPS II at admission	–	24 [21–26]	52 [49–57]	53 [46–57]
PaO_2_/FiO_2_,^a^ mmHg	–	145 [130–201]	220 [202–223]	105 [98–122]
PEEP,^a^ mmHg	–	5 [5–7]	5 [5–7]	8 [8–12]
Lung Injury Score^a^	–	–	1.5 [1.25–1.75]	2.25 [2–2.25]
Causes of ARDS/ALI, *n*				
Direct	–	–	6	8
Pneumonia			5	7
Gastric inhalation			1	1
Indirect	–	–	3	5
Pancreatitis			2	2
Intra-abdominal infection			1	3

*ACPE* acute cardiogenic pulmonary edema, *ALI* acute lung injury, *ARDS* acute respiratory distress syndrome, *FiO*_*2*_ fraction of inspired oxygen, *PaO*_*2*_ partial pressure of oxygen in arterial blood, *PEEP* positive end-expiratory pressure, *SAPS II* Simplified Acute Physiology Score II

Results are expressed as median [IQR] unless otherwise indicated

^a^Lower PaO_2_/FiO_2_ ratio during the first 48 h

### Serum of patients with ARDS induced leukocyte stiffening

**Fig. 1:**
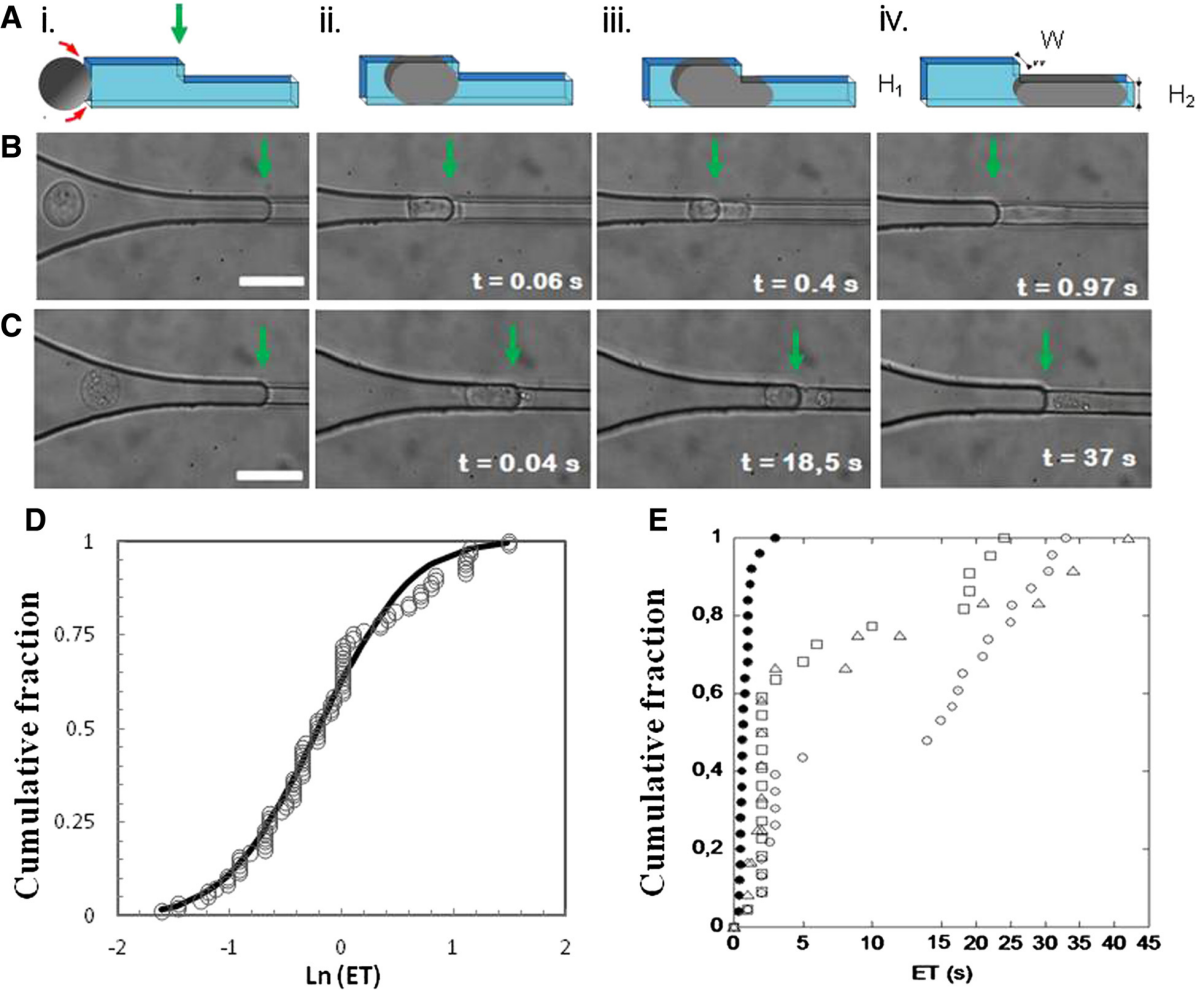
Microfluidic assay of leukocyte stiffness. **a**–**c** Leukocyte (THP-1) entering the microfluidic stiffness tester (**a**) represented on a schematic and (**b**) and (**c**) observed by video microscopy for a local suction pressure ΔP = 160 Pa, and after 1-h incubation with the serum (**b**) of a healthy volunteer and (**c**) of a patient with moderate to severe acute respiratory distress syndrome (ARDS). The green arrows indicate the entry of constriction C2. (i) Cell entering the funneled constriction C1, (ii) early cell contact with the entrance of constriction C2, (iii) the development of a cell projection in constriction C2, and (iv) completed entry in constriction C2. The *scale bar* represents 20 μm, the timing origin corresponds to first contact of the cell with entry of C2, and the entry time (ET) corresponds to the time for completed entry of a cell in C2 (see movie 1 in Additional file 2). **d** and **e** Cells stiffened by sera of patients with ARDS are distributed in two populations. Cumulative fraction of ET at ΔP = 160 Pa for THP-1 cells (**d**) incubated in serum of a healthy donor (*filled circles*) with a fit by a log-normal (Ln) distribution with μ = 0.8 and σ^2^ = 3.7 s^2^ (*black line*) and (**e**) incubated with the sera of three patients with moderate to severe ARDS (*open symbols*). All cells are stiffened and distributed in a bimodel population of moderately and highly stiffened cells. For each patient serum sample, the numbers of tested cells were 250 in (**d**) and 50 in (**e**)

**Fig. 2:**
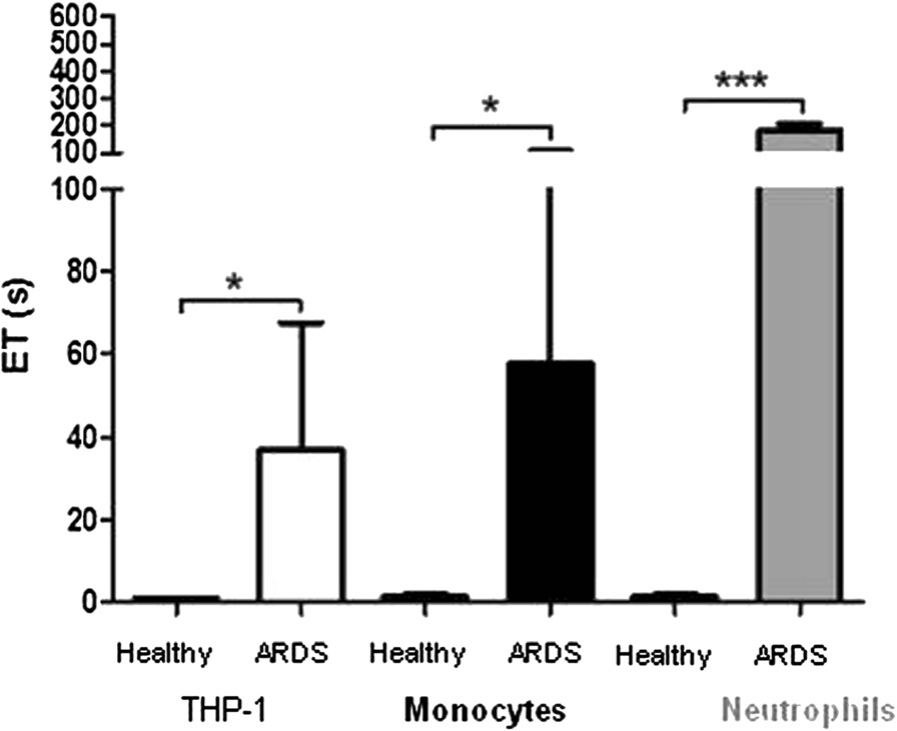
Serum of a patient with acute respiratory distress syndrome (ARDS) induced stiffening on primary monocytes and neutrophils. Entry times (ETs) measured for THP-1 cells (*open bar*), primary monocytes (*black bars*), and primary neutrophils (*gray bars*) after 1-h incubation with the sera of a healthy donor and of a patient with ARDS. The number of tested cells per condition was >30. Values are mean ± standard deviation. *p < 0.05; ***p < 0.0001 (Mann-Whitney *U* test). For neutrophils incubated in serum of patients with ARDS, cells never completed their entry in C2 at 300 seconds and experiments were stopped. ETs are reported as >300 seconds, and there is no error bar

A typical measurement with the microfluidic stiffness tester is presented in Fig. 1a-c (see Additional file 2 for an additional movie file showing this in more detail). THP-1 cells incubated with healthy serum (Fig. 1d) were characterized by a log-normal distribution, whereas ARDS serum incubated THP-1 cells (Fig. 1e) had greatly widened distributions with two populations of roughly similar size: one population of moderately stiffened cells, with ETs one order of magnitude greater than healthy cells (2 < ET < 5 seconds), and another population of highly stiff cells with ETs up to two orders of magnitude greater than healthy cells (15 < ET < 100 seconds). This double population distribution was a general feature of ARDS serum incubated cells. In primary fresh neutrophils and monocytes, as in THP-1 line cells (Fig. 2), sera of patients with ARDS induced rapid and intense stiffening, showing that the stiffening effect of the sera of patients with ARDS acted on all leukocytes.

### Leukocyte stiffening was associated with ARDS severity

**Fig. 3:**
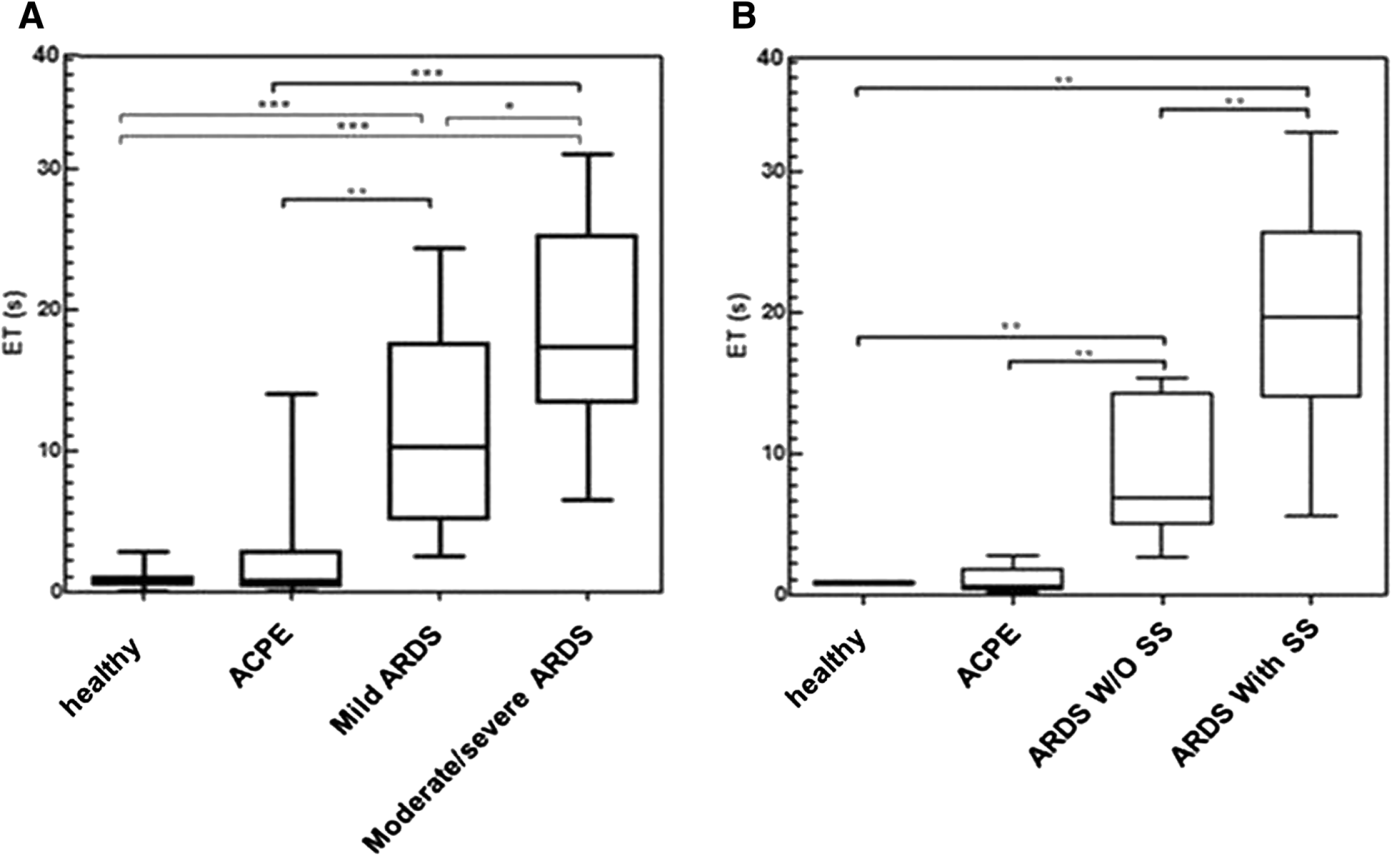
**a** Stiffness vs. lung injury. Distributions of the median entry times (ETs) measured after 1-h incubation of THP-1 cells with the sera of healthy donors (control; *n* = 5), patients with acute cardiogenic pulmonary edema (ACPE; *n* = 6), patients with mild acute respiratory distress syndrome (ARDS; *n* = 9, among whom 5 had septic shock [SS]), and patients with moderate to severe ARDS (*n* = 13, among whom 10 had septic shock). **b** Stiffness vs. SS. Distributions of the median ETs measured after 1-h incubation of THP-1 cells with the sera of healthy subjects (*n* = 5), patients with ACPE (*n* = 6), patients with ARDS but without septic shock (*n* = 7), and patients with both ARDS and septic shock (*n* = 15). The number of tested cells per patient was 50. Box plots represent the median (*black bar inside box*), interquartile range (*box*), and minimum and maximum values (*whiskers*). *p < 0.05; **p < 0.0025; ***p < 0.0001 (Mann-Whitney *U* test)

Compared with the sera of healthy subjects, the sera of patients with ACPE did not affect THP-1 stiffening (Fig. 3a), whereas the sera of patients with mild or moderate to severe ARDS increased the ETs of THP-1 cells. Hence, the sera of patients with ARDS induced leukocyte stiffening, whereas cardiogenic edema was not a relevant parameter in this process. This finding supports the hypothesis that leukocyte stiffening induced by serum is related to the severity of lung injury due to ARDS.

### Leukocyte stiffening effects were associated with lung injury

To evaluate the respective roles of ARDS and SS, we compared the ETs between patients presenting with ARDS without SS and patients presenting with ARDS with SS. As shown in Fig. 3b, the group of patients presenting with ARDS with SS had higher ETs than the group presenting with ARDS without SS. However, the group of patients with ARDS and without SS showed significantly higher ETs than the healthy subjects and patients with ACPE (*p* < 0.0025). This finding demonstrates a specific link between ARDS and leukocyte stiffening, independent of the presence of SS.

### IL-1β–, IL-8–, and TNF-α–induced leukocyte stiffening

**Fig. 4:**
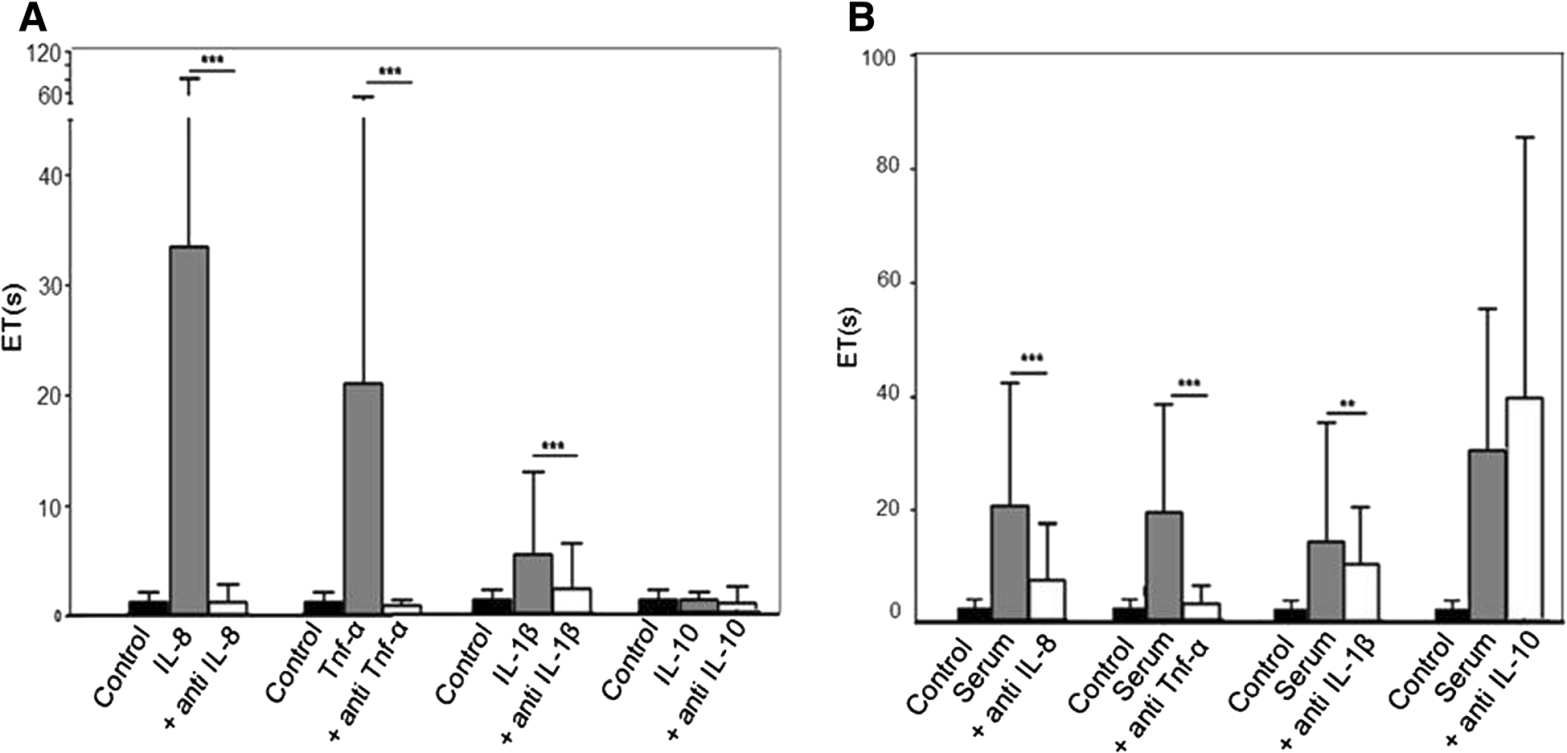
**a** The effect of cytokine recombinants on THP-1 stiffness. Entry times (ETs) measured after 1-h incubation of THP-1 cells with interleukin (IL)-8, tumor necrosis factor (TNF)-α, IL-1β, and IL-10 at 1000 pg/ml, 20 pg/ml, 10 pg/ml, and 200 pg/ml, respectively, without (*gray bars*) or with (*open bars*) 1 μg/ml anti-IL-8, anti-TNF-α, anti-IL-1β, and anti-IL-10 antibodies (Ab), respectively. The *black bar* represents control cells without cytokines and Ab. **b** The effect of serum of patients with acute respiratory distress syndrome (ARDS) with blocking Ab of IL-8, TNF-α, and IL-1. ETs measured after 1-h incubation of THP-1 cells with the serum of patients with ARDS, without (*gray bars*) and with (*open bars*) 1 μg/ml of anti-IL-8, anti-TNF-α, anti-IL-1β, and anti-IL-10 Ab. The *black bar* corresponds to control cells without patient serum and Ab. The number of tested cells per incubation condition was 50. Values are the mean (*top of box*) ± standard deviation (*whiskers*). **p < 0.0025; ***p < 0.0001 (Student’s *t* test)

The serum levels of cytokines are reported in Additional file 1: Table S1. We found positive correlations between the ETs and the cytokine levels of IL-1β, IL8, IL-10, and TNF-α (see Additional file 1: Figure S2). Figure 4a shows that recombinant IL-1β, IL-8, and TNF-α (at the maximum concentration found in the patient sera) each induced a pronounced stiffening of THP-1 cells, whereas IL-10 had no significant effect. Furthermore, incubation of THP-1 cells with recombinant IL-1β, IL-8, or TNF-α and with a large excess of blocking Ab anti-IL-1β, anti-IL-8, or anti-TNF-α did not affect cell stiffness compared with that of the controls without cytokines and Ab.

### Partial reversal of serum-induced stiffening by antibodies against IL-1β, IL-8, and TNF-α

We incubated THP-1 cells with sera from patients with ARDS and blocking Ab against cytokines (Fig. 4b). An excess of blocking Ab against IL-1β, IL-8, and TNF-α markedly reduced serum-induced stiffening, whereas the blocking Ab against IL-10 had no effect. These results proved that IL-1β, IL-8, and TNF-α participated significantly in the leukocyte stiffening effect induced by the sera of patients with ARDS. Nevertheless, cells protected by the three blocking Ab remained significantly stiffer than the control cells, suggesting that IL-1β, IL-8, and TNF-α were not the only factors in the stiffening process induced by sera or that synergistic effects might exist. Mixtures of IL-8 and TNF-α at 1000 pg/ml and 20 pg/ml, respectively, did not induce significant synergistic effects compared with isolated components.

### Role of adhesion

**Fig. 5:**
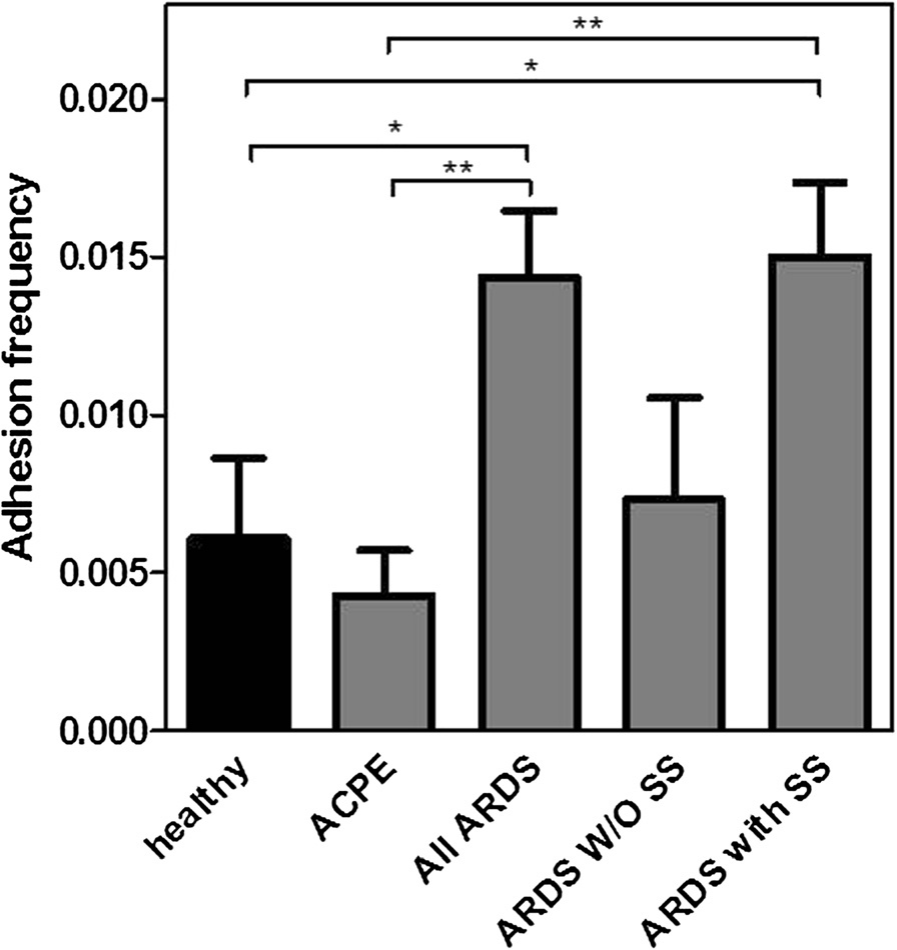
Leukocyte–endothelium adhesion. The frequency of arrest per unit length of THP-1 cells flown at a shear rate of 9.6/second on top of a confluent human umbilical vein endothelial cell layer after 24-h incubation with the sera of healthy donors, patients with acute cardiogenic pulmonary edema (ACPE; *n* = 6), patients with acute respiratory distress syndrome (All ARDS; *n* = 22), patients with ARDS but without septic shock (ARDS W/OSS; *n* = 7), and patients with both ARDS and SS (*ARDS with SS; n* = 15). Values are mean ± standard error of the mean. The number of tested cells was >200 per patient. *p < 0.05 ; **p < 0.0025 (Student’s *t* test)

Figure 5 presents the adhesion frequency of THP-1 cells on monolayers of endothelial cells. There were no detectable changes in adhesion frequencies for ACPE and ARDS without SS, whereas an increase in the frequency was observed in ARDS with SS. Adhesion measurements under flow suggested that the increased adhesion was related to SS rather than to ARDS.

### Integrin receptor expression by incubations in patient sera is nonsignificant

**Fig. 6:**
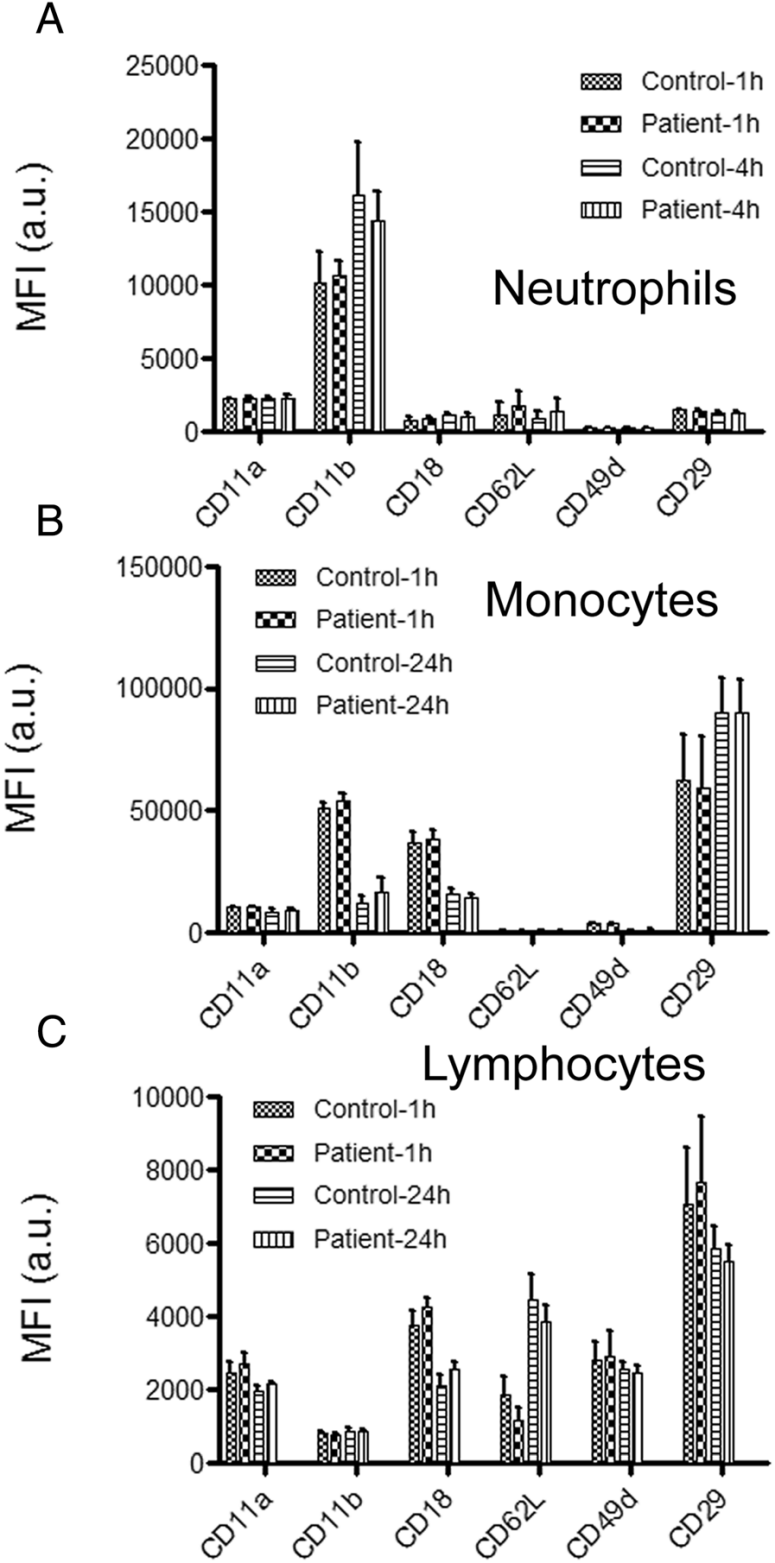
The expression of adhesion molecules. Cytometry data for human (**a**) primary neutrophils, (**b**) monocytes and (**c**) lymphocytes. The graph report the (MFI) for CD11a, CD11b, CD18, CD62L, CD49d, CD29 measured with healthy donors and ARDS patient samples at 1 and 4h for neutrophils, and at 1 and 24 h for monocytes and lymphocytes. The number of samples for healthy donors (n = 10) and patients with ARDS (n = 22). MFI values are the mean +/- SD. Mann-Whitney p-values are all > 0.05 between healthy donors and ARDS patients

The expression of surface receptors (CD62L, CD11a, CD11b, CD18, and CD49d) was found to be roughly similar for the groups of healthy donors and patients with ARDS after incubations for 1 h and 24 h for lymphocytes and monocytes, and for 1 h and 4 h for neutrophils (Fig. 6). Even sera of patients with SS did not induce significant upregulation of surface receptors at the same concentration of 10 % serum in RPMI medium that was also used for the stiffening experiments.

### Actin cytoskeleton and stiffening

The incubation of cells with the actin polymerization inhibitor latrunculin A was found to inhibit serum-induced stiffening to a great extent, whereas the tubulin polymerization inhibitor nocodazole had a negligible impact (see Additional file 1: Figure S3). The results suggested that cell stiffening was actin-dependent.

## Discussion

In the present study, we have shown that the serum of patients with ARDS induced rapid and intense actin-dependent stiffening of leukocytes. This leukocyte stiffening was associated with the severity of ARDS. The low expression of adhesion molecules of the integrin family suggested that leukocyte stiffening plays a more important role than adhesiveness in their sequestration in the lung microvasculature in the early phase of ARDS. Analysis of serum composition and the use of recombinant cytokines showed that cytokines IL-1β, IL-8, and TNF-α were major contributors to leukocyte stiffening by the sera of patients with ARDS. Finally, blocking Ab against IL-1β, IL-8, and TNF-α was found to protect leukocytes from serum-induced stiffening.

Various methods have been employed to evaluate leukocyte deformability, but there is no standard tool for clinical applications. Microfiltration experiments, such as cell transit analysis, have been used to study sepsis and lung injury [10, 18], but the transit times of cell suspensions through micropore filters remain qualitative and cannot distinguish between the effects of adhesiveness or stiffness, because both are possible in these assays. Conversely, single-cell methods, such as micropipette aspiration [19], cell poker [15, 25], or atomic force microscopy [14], allow for precise mechanical measurements, but their throughput is too low for medical studies. The strategy of using microfluidics allows one to design higher-throughput experiments [14, 25] for adhesiveness or stiffness and to mimic the targeted physiological conditions of the geometry (approximately 6 μm) and pressure (about 100 Pa) of the lung microvasculature, as was done in the present study.

We chose to study the stiffening of leukocytes using THP-1 cells, mainly because this cell line, but not freshly isolated primary neutrophils, allows for the establishment of a stable and reproducible analysis protocol. The relevance of using monocytes and a cell line to analyze ARDS mechanisms is arguable. However, researchers in some studies have reported evidence of neutrophil-independent mechanisms of ARDS [28, 29], supporting nonnegligible roles of other types of leukocytes and suggesting that monocytes indeed contribute substantially to neutrophil recruitment in lungs during pulmonary inflammation and injury in mouse models [30, 31]. Additionally, the THP-1 cell line as a model was supported by the strong similarities of these cells to monocytes with regard to migration stimulated by *N*-formylmethionyl-leucyl-phenylalanine [32], phospholipase D activation of adhesion [33], increase in the mechanical strength of adhesion to endothelial cells induced by monocyte colony-stimulating factor [34], stimulation with coronavirus responsible for an ARDS-like syndrome [35], and the binding of chemotactic factors to THP-1 cells and neutrophils [36]. Finally, we showed that the stiffening effects of the sera of patients with ARDS were ubiquitous on THP-1 cells, monocytes, and neutrophils.

Abnormal leukocyte stiffness has been reported in previous studies of SS and ARDS [4, 6, 10–12, 14, 18], but our data provide the first evidence, to the best of our knowledge, that leukocyte stiffening is an intrinsic characteristic of ARDS, independent of the presence of SS or cardiogenic pulmonary edema. To the best of our knowledge, the investigators in the only study in which leukocyte stiffness during ARDS was measured [12] reported that leukocyte populations were moderately stiffened and that very few cells (<5 %) had extremely high stiffness. We also found a bimodal distribution, but interestingly the population of highly stiff cells represented, in our data, approximately 50 % of all of the cells. This finding suggested that the earlier study of patients’ cells [12] could have been biased by the trapping of the stiffest cells in the patients’ microvasculature, whereas our serum-based findings, with a large proportion of more stiffened cells, would be more representative of patients’ whole leukocyte populations. Our study is also the first to demonstrate leukocyte stiffening induced by incubation with the sera of patients with ARDS. The serum stiffening effect was rather rapid (<1 h), compatible with a role in the early triggering of leukocyte sequestration in the microvasculature. In the end, the associations of the severity of ARDS with leukocyte stiffening and the presence of leukocyte stiffening independent of SS support an intrinsic role for leukocyte stiffness in the pathogenesis of lung injury.

Regarding leukocyte adhesion evaluated by the expression of integrin receptors, the literature shows a very limited and rather slow upregulation of adhesion molecules in the plasma of patients with sepsis (e.g., CD49d for neutrophils) [8–10]. With our samples and with similar incubation conditions, faint effects of adhesion molecule upregulation were not detectable, whereas cell stiffening was found to be fast and strong. This finding suggests that circulating factors might favor leukocyte adhesion, but only after a delay of several hours. Interestingly, adhesion, as shown by laminar flow experiments after exposure to ARDS sera, was not significantly increased, further demonstrating that adhesion might not play a major role in early ARDS. In the end, one could conclude that leukocyte arrest in the microvasculature in ARDS might be triggered by rapid leukocyte stiffening at early time points (within 1 h), whereas adhesiveness may develop at later stages. Interestingly, similar scenarios have previously been proposed for other pathologies [37–39].

Increased IL-6 was reported in patients with sepsis and leukocyte sequestration [9], as in our data. However, we found no correlation between IL-6 and leukocyte stiffening, suggesting that the role of IL-6, if any, is not dominant. Indeed, leukocyte stiffening appeared in vitro at IL-6 concentrations >10,000 pg/ml [20], which is much higher than the content in the sera of our patients with ARDS. We found that TNF-α, IL-1β, and IL-8 induced leukocyte stiffening. Similar effects were reported for TNF-α and IL-1β at concentration thresholds >100 pg/ml [13, 20]. We showed a significant stiffening effect at <10 pg/ml for IL-1β and at 20 pg/ml for TNF-α. For IL-8, previous researchers have reported no effects [13, 20] at 10,000 pg/ml and a transient (1 h) effect at 1000 pg/ml [17]. We found strong stiffening within 1 h with IL-8 at only 1000 pg/ml. Our experiments with Ab confirmed the direct participation of these three cytokines in the stiffening effect of serum, as monoblockade of IL-1β, IL-8, or TNF-α significantly reduced the effect. However, our results with pure recombinants and with sera mixed with Ab against each cytokine suggested that other substances, which remain to be identified, played additive and/or synergistic roles.

There is not yet any therapeutic treatment to reverse abnormal leukocyte stiffening. Cytochalasin D is efficient but has a systemic toxicity. Pentoxifylline, a phosphodiesterase inhibitor, reduces neutrophil stiffening, but its efficacy for treating hemorrhagic shock has been found to be very limited [14, 40]. Blocking Ab against TNF-α provided weak prophylactic and poor therapeutic effects in large cohorts of patients with sepsis [37]. Nevertheless, patients with sepsis and lung injury might benefit from the blockade of multiple cytokine pathways because researchers in recent studies of mice and humans have reported beneficial responses in cases of acute lung injury [38] and sepsis [39]. Our results regarding protection against stiffening by blocking Ab support the exploration of cytokine regulation for therapeutic purposes. Previous unsuccessful clinical assays have been based on long-term treatments, and future assays could benefit from regular monitoring of leukocyte stiffness during treatment with a microfluidic tool to adjust the treatment dose and time. This rationale would also be helpful for other therapeutic approaches, such as treatment with sivelestat, a neutrophil elastase inhibitor that limited leukocyte stiffening in vitro [18], benefited pulmonary function [41], and improved the survival of patients with ARDS with sepsis [42].

## Conclusions

We have shown that the serum of patients with ARDS induced rapid and intense actin-dependent stiffening of leukocytes. This leukocyte stiffening was associated with the severity of ARDS. The low induction by patient sera of adhesion to endothelial cells and of upregulation of adhesion molecules suggested that leukocyte stiffening plays an important role in their sequestration in the lung microvasculature in the early phase of ARDS. Stiffening was in large part induced by increased levels of IL-1β, IL-8, and TNF-α in the sera of patients with ARDS. Ab against these three cytokines protected leukocytes from the serum-induced stiffening.

## Key messages

- The sera of patients with ARDS induced rapid and intense actin-dependent leukocyte stiffening.
- The relationship between leukocyte stiffening and the severity of ARDS suggests a role for leukocyte stiffening in ARDS pathophysiology.
- Stiffness, rather than adhesiveness, might play an important role in leukocyte sequestration in early ARDS.
- IL-1β, IL-8, and TNF-α are major contributors to leukocyte stiffening in ARDS sera.
- Blocking Ab against IL-1β, IL-8, and TNF-α was shown to protect leukocytes from stiffening in sera.

## Additional files

Additional file 1: **Online supplemental files containing Table S1, Figure S1, Figure S2, and Figure S3.** (DOCX 298 kb)

Additional file 2: **Movie 1: The cell-stiffening effect of ARDS patient serum revealed by microfluidic tests.** Bright-field video microscopy sequences of THP-1 cells pushed into a microfluidic constriction with a cross-section of 6 μm × 9 μm at an applied pressure *ΔP*_*ext*_ = 400 Pa. The cell was incubated for 30 minutes with the serum (at 10 %) of a healthy volunteer (first sequence) and of a patient with ARDS (second sequence). Videos are displayed at actual speed. (WMV 9532 kb)

## Abbreviations

Ab: antibodies
ACPE: acute cardiogenic pulmonary edema
ALI: acute lung injury
ARDS: acute respiratory distress syndrome
ELISA: enzyme-linked immunosorbent assay
ET: entry time
FiO_2_: fraction of inspired oxygen
FSC-A: forward scatter area
ICU: intensive care unit
IFN-γ: interferon-γ
IL: interleukin
IgG1: immunoglobulin G1
MFI: mean fluorescence intensity
PaO_2_: partial pressure of oxygen in arterial blood
PBMC: peripheral blood mononuclear cell
PEEP: positive end-expiratory pressure
SAPS II: Simplified Acute Physiology Score II
SS: septic shock
SSC-A: side scatter area
TNF-α: tumor necrosis factor-α
