# Supplementary material for: The leukocyte-stiffening property of plasma in early acute respiratory distress syndrome (ARDS) revealed by a microfluidic single-cell study: the role of cytokines and protection with antibodies
Source: Crit Care. 2016 Jan 12;20:8. doi: 10.1186/s13054-015-1157-5 (PMC4711060; doi:10.1186/s13054-015-1157-5)
Supplement: Supplementary file 1 — Online supplemental files containing Table S1, Figure S1, Figure S2, and Figure S3. (DOCX 298 kb) [file 13054_2015_1157_MOESM1_ESM.docx]

**ONLINE SUPPLEMENTAL MATERIAL**

**Supplementary Table**

|  | IL-1β (pg/ml) | IL-6 (pg/ml) | IL-8 (pg/ml) | IL-10 (pg/ml) | IL-17 (pg/ml) | TNFα (pg/ml) | IFN-γ (pg/ml) |
| --- | --- | --- | --- | --- | --- | --- | --- |
| ***Healthy (n=5)*** | <1 | 1.1±0.9 | <3.5 | 0.55±5.1 | <15 | - | 43.3±26 |
| ***ACPE (n=6)*** | 1.1±1.1 | 28±22 | - | 19.5±28 | <15 | 1.6±1.8 | - |
| ***Mild ARDS (n=9)*** | 1.5±1.4 | 11.8±2.1 | 16.4±264 | 7±40.6 | <15 | 2.6±1.6 | 11.2±10 |
| ***Moderate/severe ARDS( n=13)*** | 2.5±1.2 | 11.8±1.6 | 151±353 | 21.8±44.7 | <15 | 1.9±2.3 | 15.7±25.7 |
|  |  |  |  |  |  |  |  |
| **Rho Spearman** | 0.50 | 0.33 | 0.81 | 0.56 | NA | 0.80 | -0.26 |
| ***P*** | 0.016 | ns | 0.0016 | 0.0026 | NA | 0.0001 | ns |

**Table S 1:** **Cytokines levels in sera and correlation between Entry Time and cytokines levels**. **(Above grey row)** Elisa measurements of cytokines IL-1β, IL-6. IL-8, IL-10, TNF-α, and IFN-γ in sera of patients groups. Data correspond to median ± SD. **(Below grey row)** Spearman's rank correlation coefficient rho and p-factor determined between the median ETs measured for each patient and the corresponding cytokine concentration measured in patients’ sera.

**Supplementary Figures**

**
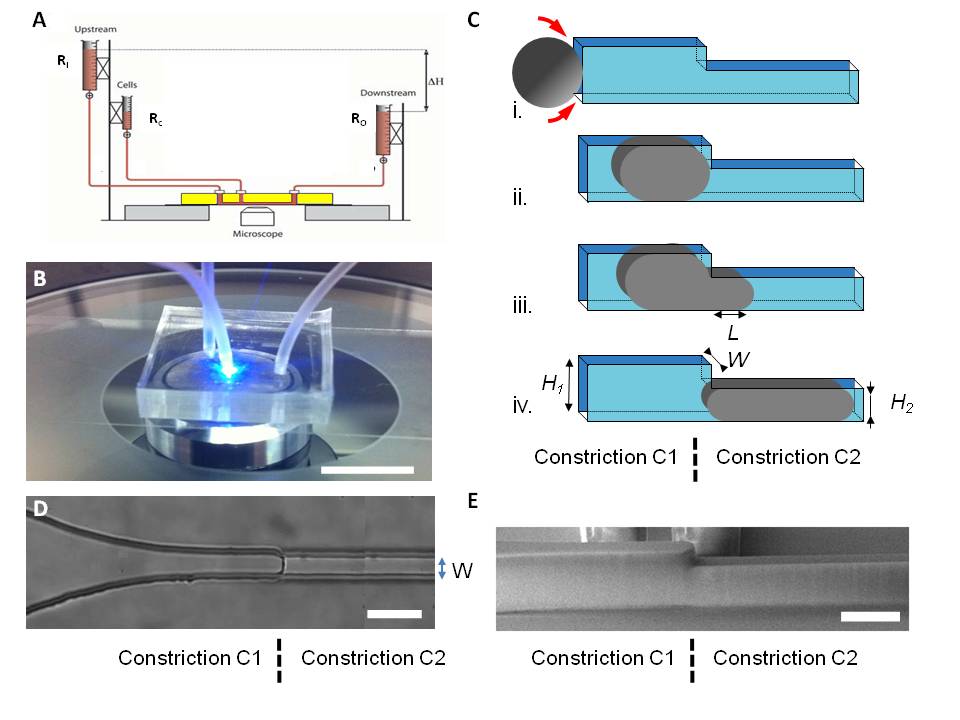
**

**Figure S 1:** **Microfluidic** **cell stiffness tester.** **(A)** Cartoon of the fluidic set-up showing the microdevice (yellow) connected to the macro-reservoirs for fluid input (*R_I_*), fluid output (*R_O_*), and cells injection (*R_C_*). Pressure drop across the device ΔP_ext_ is set by changing the height *ΔH* of *R_I_* relatively to *R_C_* and *R_O_*. **(B)** Picture of a microfluidic device on a microscope stage with tubing coming from fluid macro-reservoirs. Scale bar corresponds to 1 cm. **(C)** Double constriction device rationale. i. Spherical cell blocked at the entrance of a rectangular constriction. Arrows schematize flow leaks around the cell in channel corners. ii. Cell squeezed in C1, its deformed shape obstructs the channel’s cross-section. iii. Cell deformation during entry into C2, *L* characterizes the length of the cell projection in C2 and iv. Cell completely entered in C2. *W*, *H_1_* and *H_2_* are respectively the width of C1 and C2, the height of C1 and the height of C2. Scale bar correspond to 10 µm. **(D)** Optical micrograph of the microfluidic constriction used to test cell stiffness. C1 and C2 have a same width *W* = 6 µm. Scale bar corresponds to 10 µm.  **(E)** Electron micrograph of a mould of constrictions C1 and C2. Scale bar correspond to 10 µm.


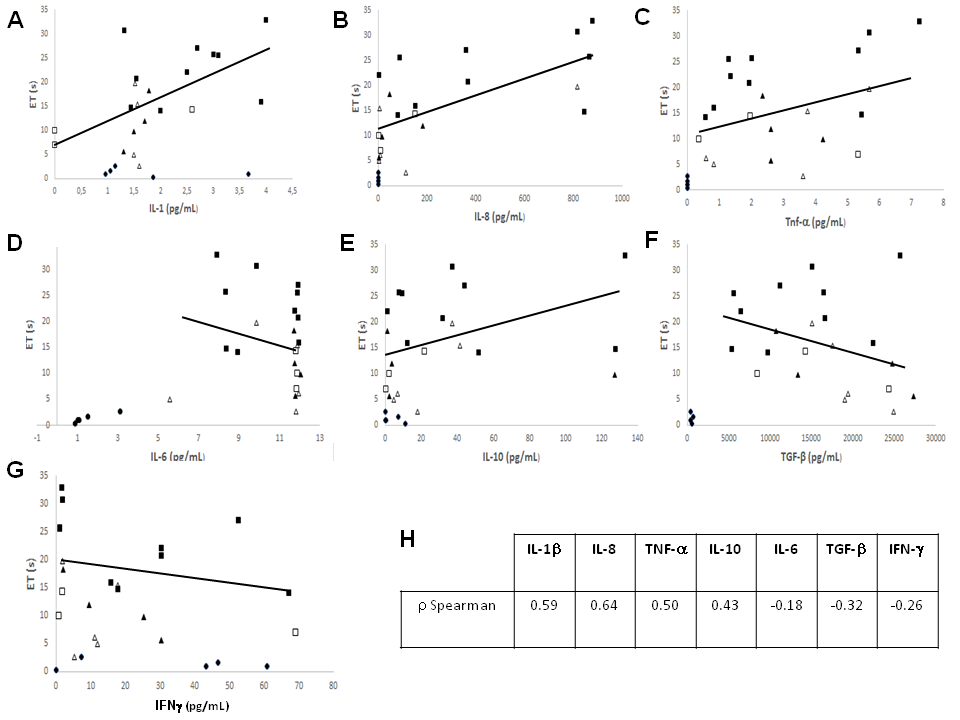


**Figure S 2: Correlation between cell stiffness and cytokine level in patient sera. (A-G)** Entrance time versus cytokine level in patient sera for IL-1β (A), IL-8 (B), TNF-α (C), IL-6 (D), IL-10 (F), and IFN-γ (G). Each symbol corresponds to one patient serum. Triangles are for mild ARDS, squares for moderate/severe ARDS, black symbols for patients who have also septic shock, and hollow symbols for patients without septic shock. **(H)** Coefficient ρ-Spearman between ET and cytokines levels for all sera tested.


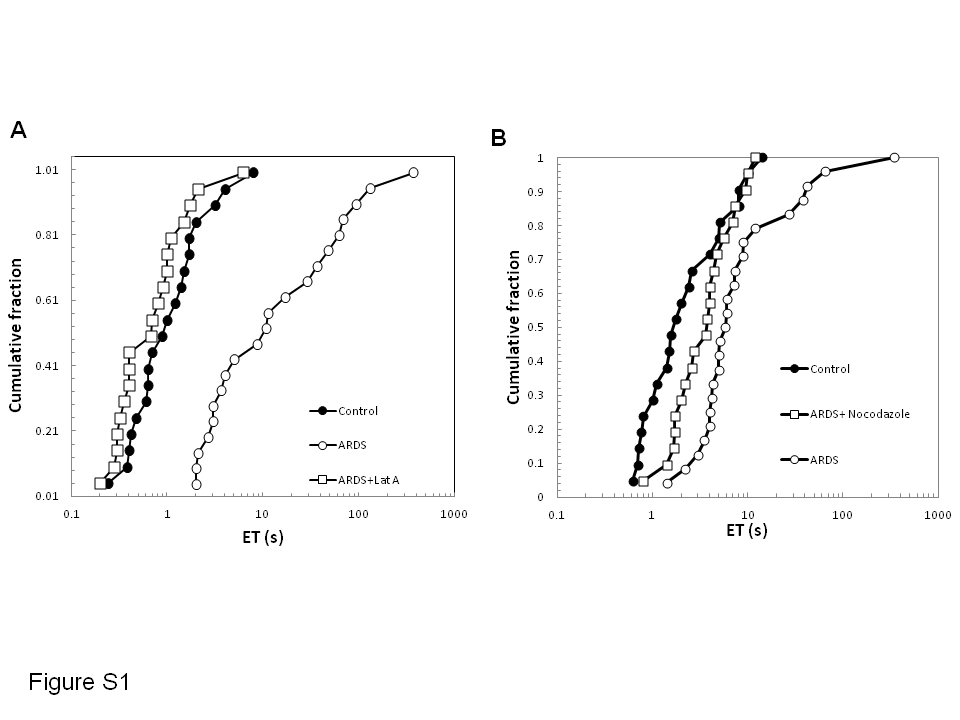


**Figure S 3:** **Cell stiffness changes are actin-dependant.** Cumulative fraction of cells versus entrance time *ET* at *ΔP* = 160 Pa for normal THP-1 cells (●), and THP-1 cells after 1h incubation in a serum of ARDS patient with (□), and without (○) addition of **(A)** Latrunculin-A at 3 µg/mL and **(B)** nocodazole at 5 µg/mL for 30 min
